# Laboratory modelling of urban flooding

**DOI:** 10.1038/s41597-022-01282-w

**Published:** 2022-04-11

**Authors:** Xuefang Li, Sébastien Erpicum, Emmanuel Mignot, Pierre Archambeau, Michel Pirotton, Benjamin Dewals

**Affiliations:** 1 College of Civil Engineering and Architecture, GuangXi University, Nanning, China; 2 Hydraulics in Environmental and Civil Engineering(HECE), University of Liège, Liège, Belgium; 3 LMFA, CNRS-Université de Lyon, INSA Lyon, Ecole Centrale de Lyon, Université Claude Bernard Lyon 1, Villeurbanne, France

**Keywords:** Hydrology, Natural hazards

## Abstract

This paper presents two datasets obtained from laboratory experiments of urban flooding in a street network performed at the University of Liège. The experimental model represents a part of a synthetic urban district that consists of three inlets, three outlets and several three- and four- branches crossroads. The following experimental data was produced: (i) dataset 1: time-series of flow depths at model inlets and time-series of discharges at model outlets for a two-branch junction model, a two-branch bifurcation model and a district model. The datasets were generated by varying the upstream and downstream boundary conditions, i.e. flooding conditions; (ii) dataset 2 includes the same data type as dataset 1 complemented by 2D surface velocity measured using the non-intrusive LSPIV technique for eight urban form configurations in the district model. The collected data enable improving the understanding of the effect of urban forms on the urban flood processes. These two datasets are valuable for validating and improving numerical or analytical models of urban flooding and may contribute to flood risk management and flood-resilient urban design.

## Abstract (summary)

**Table:** 

Measurement(s)	flow depth • Outflow discharge • Surface velocity field • Inflow discharge
Technology Type(s)	Ultrasonic sensor • Calibrated rating curve *Q~H* • Large Scale Particle Images Velocity (LSPIV) with a commercial camera LUMIX-GH4 • Electromagnetic flowmeters
Factor Type(s)	Flooding scenario • Model geometry • Scale factors
Sample Characteristic - Organism	Water
Sample Characteristic - Environment	laboratory environment
Sample Characteristic - Location	Belgium

## Background & Summary

Urban flood risk is increasing worldwide due to changes in the human and natural environments, such as fast urbanization^1^ and more frequent extreme rainfall events^2^. Hydrological and hydraulic modelling software are powerful tools for predicting, analyzing and managing urban flood risk^3,4^. However, the applicability or accuracy of these computational models for simulating flow in urban areas remains hampered by a lack of adequate validation data^4,5^, which should include not only accurate estimations of flood extents, but also the spatial distribution of flow depths, discharge partition in-between the streets as well as flow velocity. All these parameters are indeed critical inputs for flood impact modelling^6^. In most cases, existing field data collected by remote sensing are limited to watermarks or pointwise flow depths. Such data are not sufficient to reflect the complexity of flow in densely urbanized floodplains characterized by multi-directional pathways. Therefore, laboratory experiments provide useful complementary information for validating computational models of flood hazard.

Mignot et al. (2009)^7^ summarized 45+ experimental studies of urban flooding and pinpointed the value of laboratory datasets for improving the performance of computational models^8^. Compared to field observations during real-world flood events, laboratory measurements enable more detailed monitoring of flow variables, under known and controlled boundary conditions and for arbitrary scenarios (even more extreme than ever experienced in the field).

Laboratory experiments of urban flooding at the district level are usually conducted based on a geometrically distorted scale model (i.e. the scale factor applied along the vertical direction is less than the scale factor applied along the horizontal direction). This stems from the large horizontal length scales characterizing urban flooding compared to the vertical scales. The geometric distortion enables reducing the relative errors in the measurements and ensures a turbulence regime in the scale model more representative of the regime at the prototype scale. However, the flow aspect ratio is altered by geometric distortion^9–11^.

The datasets presented here complements analyses detailed in two recent papers by Li et al. (2021, 2021a)^9,12^. The original contributions of the present datasets in these studies are twofold:

(i) Quantify for the first time the effect of model geometric distortion on flow characteristics in laboratory models of urban flooding at the district level^9–11,13^ (Dataset 1). Measurements of flow depths at street inlets and flow discharge at street outlets were carried for three layouts of streets: a junction, a bifurcation and a simplified urban district. The dataset contains observations corresponding to two flooding scenarios for the junction model (19 tests), five flooding scenarios for the bifurcation model (51 tests), and two flooding scenarios for the district model (22 tests). Details are provided in Section ‘Data Records’.
(ii) Validate the performance of computational models to investigate the influence of urban forms (i.e. the layout of buildings) on urban flooding^12^ (Dataset 2). Eight geometric configurations, corresponding to synthetic, contrasting urban forms, were tested in the laboratory model. For each urban configuration and one flooding scenario, the following flow characteristics were measured: flow depth, outflow discharge and surface velocity.

## Methods

### Experimental model

**Fig. 1:**
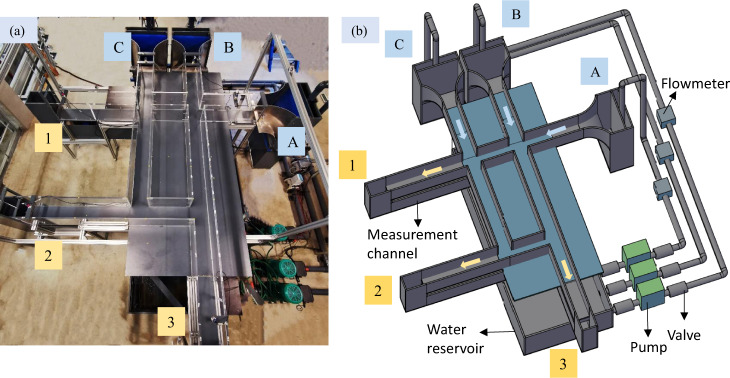
Physical model of the street network. Letters A to C and numbers 1 to 3 denote the inlets and the outlets of the physical model, respectively.

**Fig. 2:**
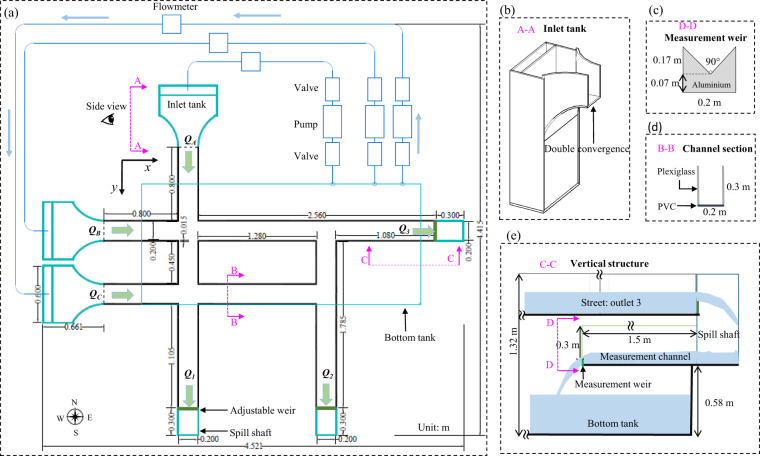
(**a**) Plan view and (**b**-**e**) details of the physical model of the street network.

The experimental facility was constructed in the laboratory of Engineering Hydraulics at the University of Liège in Belgium (Fig. 1). The physical model represents a part of an urban district, which includes four crossroads with three or four branches each (Fig. 2). The bottom is horizontal and made of smooth PVC, while the sidewalls of the streets are made of Plexiglas. The roughness height of these two materials was estimated at 5 × 10^−5 ^m. The height of the street sidewalls is 0.3 m, with a width set to 0.2 m. This corresponds to a typical street width of 10 m at prototype-scale if a horizontal scale factor *e*_*H*_ = 50 is considered (*e*_*H*_ is the ratio between the horizontal dimensions at prototype-scale and at model scale). The flow in the physical model is steady. Water is recirculated through a closed loop consisting of three independent pipes supplying water to the three street inlets, three measurement channels connected to the street outlets and a reservoir of 2.4 m^3^ located underneath the physical model (Figs. 1 and 2).

### Test design

Two complementary datasets are presented here.

#### Dataset 1

**Fig. 3:**
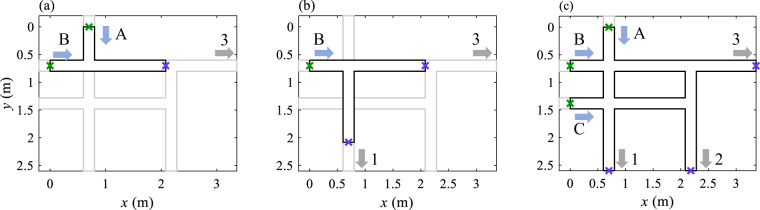
Layouts of the three experimental setups: (**a**) junction; (**b**) bifurcation; (**c**) district model. Upstream flow depths were measured at the positions shown by green crosses.

For generating the first dataset, we considered three different geometric configurations, as shown in Fig. 3:

- a junction, with two inlets and one outlet (Fig. 3a);
- a bifurcation, with one inlet and two outlets (Fig. 3b)
- and a district model, with three inlets and three outlets (Fig. 3c).

The junction and bifurcation models were created by blocking some streets of the district model. In the junction and bifurcation cases, the length of the streets upstream and downstream of the single crossroad were set, respectively, to 0.6 m and 1.28 m. The flow depths were prescribed as boundary conditions at the outlet of each street (i.e. at the positions shown by the blue cross in Fig. 3).

To investigate the effect of model geometric distortion on the observed flow variables, we defined several prototype-scale flooding scenarios, and each of them was reproduced at the model scale using a range of vertical scale factors *e*_*V*_ (i.e. the ratio between vertical dimensions at the prototype-scale and the model scale)^9^. At least two different flooding scenarios were tested for each geometric distortion value by varying the inflow discharges and/or the flow depths at the model outlets.

For the three geometric configurations, upstream and downstream flow depths were measured at positions depicted in Fig. 3 by green and blue crosses, respectively), while the discharge partition between the street outlets was measured for the bifurcation and district cases.

#### Dataset 2

**Fig. 4:**
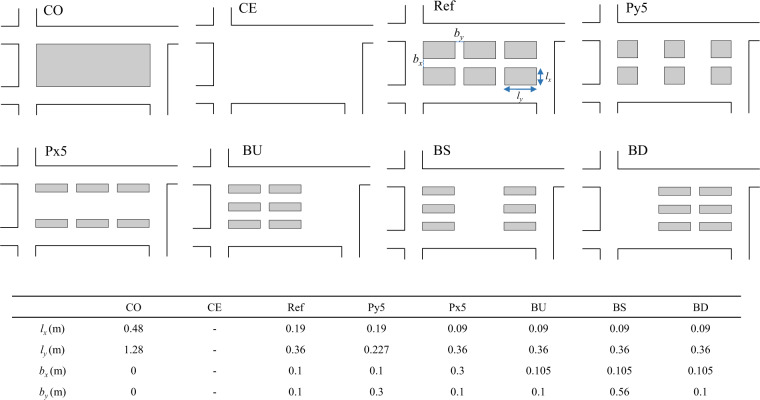
Considered urban configurations and corresponding geometric parameters *b*_*x*_ and *b*_*y*_ (minor street width), as well as *l*_*x*_ and *l*_*y*_ (building width and length). Configuration CO corresponds to the district displayed in Fig. 3c.

A second dataset was created to investigate the influence of urban forms on flow characteristics. As shown in Fig. 4, eight urban configurations were considered in the central rectangular area of the district model. One configuration (labelled CO) is simply the same as the “district” configuration in Dataset 1 (Fig. 3c). In another extreme configuration (labelled CE), the central area is assumed empty. A reference configuration (labelled Ref) was defined by including one minor street along direction *x* and two minor streets along direction *y*. In this configuration, the width of the minor streets was set to 0.1 m, i.e. half of the width of the main streets connecting the inlets to the outlets. Two additional configurations (labelled Px5 and Py5) were generated by expanding the width of the “minor” streets along the corresponding direction (*x* and *y* respectively) to 0.3 m (instead of 0.1 m). Based on the configuration Px5, three other configurations were generated by varying the location of buildings (labelled BU, BS, BD) The corresponding geometric parameters (minor street widths *b*_*x*_ and *b*_*y*_, as well as building dimensions *l*_*x*_ and *l*_*y*_) are presented in Fig. 4. For these five configurations, we investigated one flood scenario, considering a vertical scale factor *e*_*V*_ = 5. The performed measurements include flow depth upstream of the main streets, flow discharge at each outlet, as well as surface velocity over the whole district.

### Measurement techniques

#### Water level measurements

Water levels were continuously recorded with ultrasonic sensors (Microsonic: Mic + 35/IU/TC). For each test, up to nine ultrasonic sensors were used simultaneously to monitor the water levels at model inlets (*h*_*A*_, *h*_*B*_, *h*_*C*_ green crosses in Fig. 3), model outlets (*h*_*1*_, *h*_*2*_, *h*_*3¨*_blue crosses in Fig. 3) and in the measurement channels (*h*_*Q1*_, *h*_*Q*2_, *h*_*Q3*_ to evaluate the outlet discharges). The signals of the ultrasonic sensors were recorded at a sampling frequency of 100 Hz for a duration of 60 s to 150 s. These signals were then processed to discard outliers. The outliers were defined as the values situated outside the interval [*V*_*mean*_ − 3σ, *V*_*mean*_ + 3σ], where *V*_*mean*_ is the time-averaged value and σ is the standard deviation.

#### Inflow discharges

Steady inflow discharges were prescribed as upstream boundary conditions. The inflow discharge provided to each street inlet was controlled by adjusting the rotation speed of the pumps and the degree of opening of a valve situated between the pump and the flowmeter (Fig. 2a). The inflow discharges (*Q*_*A*_*, Q*_*B*_, and *Q*_*C*_) were monitored by three electromagnetic flowmeters (SIEMENS-MAG 5100 W) with an accuracy of 0.5%. The relations between the discharges and the electric output signals provided by the flowmeters were determined by calibration. During each measurement, the signals delivered by the three flowmeters were recorded simultaneously and they were processed by exactly the same methods as those used for the water level measurements.

#### Outflow discharges

**Fig. 5:**
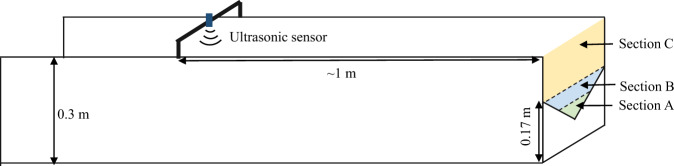
Sketch of measurement channels and weir.

The outflow discharges *Q*_1_, *Q*_2_ and *Q*_3_ were estimated with 90° triangular sharp-crested weirs placed at the downstream end of each horizontal measurement channel (Fig. 5). The rating curves of the weirs, providing the flow discharge *Q* as a function of the head *H* in the channel, were determined experimentally and approximated by a third-order polynomial:

1 $$Q=a{H}^{3}+b{H}^{2}+cH$$

with

2 $$H=h-{z}_{wc}+\frac{{v}^{2}}{2g}=h-{z}_{wc}+\frac{{Q}^{2}}{{(wh)}^{2}2g}$$

where the flow discharge *Q* is in m^3^s^−1^; the head *H* is estimated at about 1 m upstream of the triangular weir; *w* = 0.2 m is the width of the measurement channels, *h* is the flow depth (in m) measured from the bottom of the measurement channel; *z*_*wc*_ = 0.07 m is the elevation of the lowest point of the triangular weir crest (Fig. 2c), also measured from the bottom of the measurement channel; *v*^*2*^/2 *g* is the velocity head (in m).

Three ranges of flow discharge were defined to improve the measurement accuracy (Fig. 5), and parameters *a*, *b* and *c* in Eq. (1) were calibrated separately in each range.

- a first rating curve was used to measure small discharges in Section A, where the flow depth was below 0.12 m;
- a second rating curve was used to measure intermediate discharges for higher flow depths, keeping the water level below the top of the triangle weir (Section B) (Fig. 5), with a flow depth between 0.12 m and 0.17 m.
- a third rating curve was applied to estimate larger discharges, for which flow depths exceed 0.17 m (Section C).

The rating curves were derived by running the model with an adjusted geometry so that a single inlet and a single outlet were active at the time. The inflow discharge, measured by an ultrasonic flowmeter, was systematically varied and the resulting flow depth in the measurement channel was recorded.

#### Surface velocity

The non-intrusive technique Large Scale Particle Images Velocity (LSPIV) is an efficient approach for surface velocity measurements in the laboratory and the field^14–17^. In this study, a commercial camera LUMIX-GH4 was placed 2 m above the bottom of the laboratory model. The applied resolution was set to 1920 × 1080 pixels and each video was recorded for at least 60 s with a sampling rate of 25 fps. Sawdust with a diameter of 1 mm – 3 mm was used as a tracer, injected at the inlets and captured at each outlet with a basket. The extent of the experimental model was covered by combining several movies recorded from three or four viewpoints, by displacing the camera horizontally (details in the document in data packages^18^).

**Fig. 6:**
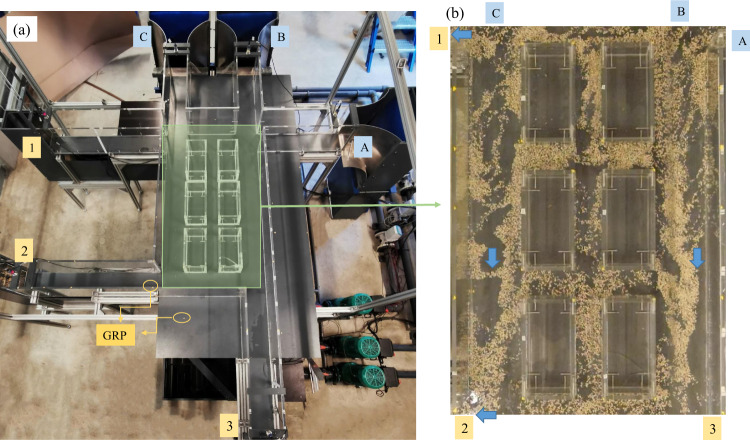
(**a**) Physical model in ‘Ref’ configuration, yellow points represent the Ground Reference Points (GRP); (**b**) a demonstration of LSPIV video.

The recorded sequences were processed with the open-source software Fudaa-LSPIV^19–21^ following three main steps to calculate the surface velocity: *(i)* a 3-D orthorectification of the images was applied based on 10 to 20 ground reference points (GRP) placed at three vertical elevations (*z* = 0 m, *z* = 0.15 m or *z* = 0.3 m, see yellow points in Fig. 6a). The spatial resolution of the corrected images is 0.001 m/pixel. *(ii)* the surface velocity was calculated by tracking the path of particles in a predefined ‘Interrogation Area’ (IA) over a selected ‘Search Area’ (SA) (details in Legout et al. (2012)^20^). *(iii)* anomalous values of calculated surface velocities were filtered out through a post-processing procedure based on threshold values for each velocity component. The time-averaged values over 60–100 s of the filtered velocity fields were retained. For each configuration, the time-averaged results obtained from each viewpoint were combined to get a single flow velocity field over the whole area of interest. More details about raw videos and video processing are provided in the data packages^18^.

## Data Records

### Data structure

Data archiving is available on the repository Zenodo^18^.

**Fig. 7:**
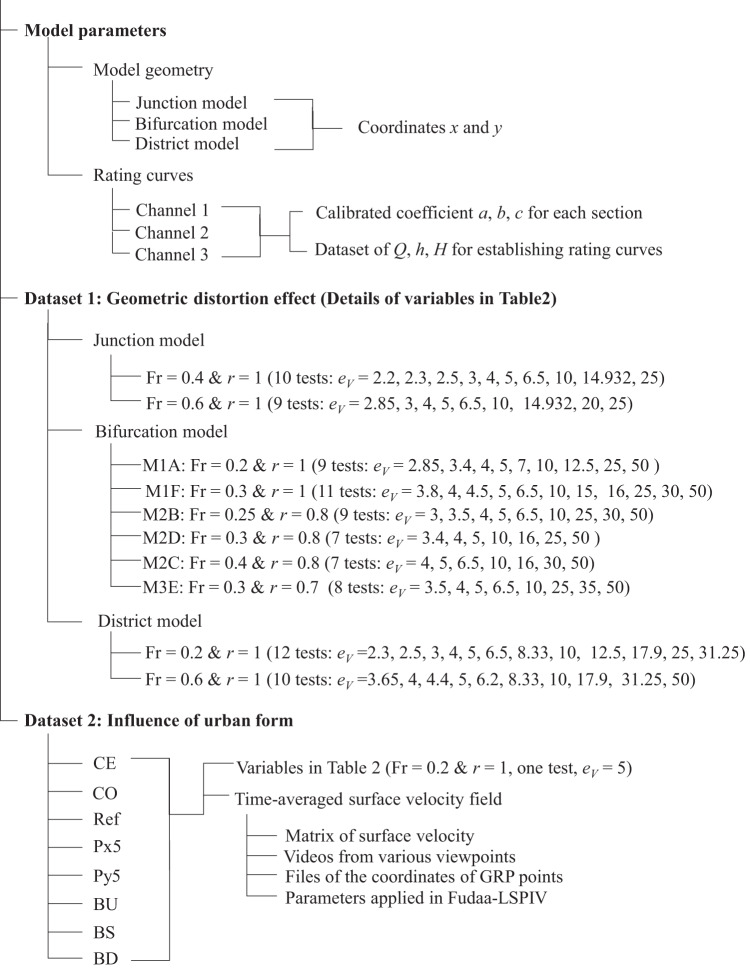
Data structure, *r* is the ratio between the flow depths at outlets 2 or 3 and the flow depth at outlet 1.

The datasets are structured as presented in Fig. 7. They include three parts: the model’s parameters, Dataset 1 and Dataset 2 as introduced in Section “Test design”. All collected data are stored in HDF5 format (Hierarchical Data Format 5) that can be read by various programming languages (e.g. *h5read* in Matlab, *h5py.File* in Python, *h5read* in R etc.).

The first part consists in the description of the experimental model (Fig. 7), including *(i)* one file *“Model_geometry.h5”* for describing the coordinates of the model geometries (i.e. junction model, bifurcation model and district model, as displayed in Fig. 3), and a second file “*ModelGeometry_data_structure.txt”* detailing the data structure; *(ii)* one HDF5 file “*RatingCurves.h5*” presenting the data used to derive the three rating curves used for computing the outlet discharges in the measurement channels, along with the calibrated coefficients as presented in Table 1; finally the data structure is detailed in the file “*RatingCurve_data_structure.txt”*.

**Table 1:** Dataset notations and units for establishing the rating curves.

Label in the dataset	Variables in Eqs. (1) and (2)		Units
a, b, c	*a, b, c*	The calibrated coefficients	(−)
Q	*Q*	Flow discharge in measurement channels	(m^3^s^−1^)
h_Q	*h*	Flow depth in measurement channels	(m)
H	*H*	Head charge in measurement channels	(m)

For the second part (Dataset 1), one HDF5 file was created for each flooding scenario in each geometric configuration (i.e. two HDF5 files for the junction, and six for the bifurcation and the district case, Fig. 7). For each flooding scenario (i.e. each HDF5 file), a series of seven to twelve tests based on various vertical scale factors were performed as presented in Fig. 7. Table 2 lists the names of all the measured variables: (i) boundary conditions, including the inflow discharges at inlets and the flow depths at outlets; (ii) measurement results, including flow depths upstream (at the inlets) and flow discharge at each outlet. Three files are available for each flooding scenario of geometric configuration:

- “<*file name*>.h5” file in HDF5 format contains all data.
- “<*file name*>_data_structure.txt” file indicates the tree structure and the detailed content of each test that are included in the “<*file name>*.h5” file. Repeated measurements are available for some scale factors. They are labelled as P2, P3, … in the test names.

**Table 2:** Dataset notations and units in the main flow.

	Label in the dataset	Variables		Units
Boundary conditions	QA, QB, QC^(1)^	*Q*_*A*_*, Q*_*B*_*, Q*_*C*_	Time-averaged inflow discharge at inlets	(m^3^h^−1^)
	QA_i, QB_i, QC_i	*Q*_*A,i*_*, Q*_*B,i*_*, Q*_*C,i*_	Temporal inflow discharge at inlets	(m^3^h^−1^)
	SD_ QA, SD_QB, SD_QC	${\sigma }_{{Q}_{A}}$, ${\sigma }_{{Q}_{B}}$, ${\sigma }_{{Q}_{C}}$	Standard deviation of inflow discharge	(m^3^h^−1^)
	h1, h2, h3	*h*_1_, *h*_2_, *h*_3_	Time-averaged flow depth at each model outlet^(2)^	(m)
	h1_i, h2_i, h3_i	*h*_1*,i*_, *h*_*2,i*_, *h*_3*,i*_	Temporal flow depth at each model outlet	(m)
	SD_h1, SD_h2, SD_h3	${\sigma }_{{h}_{1}}$, ${\sigma }_{{h}_{2}}$, ${\sigma }_{{h}_{3}}$	Standard deviation of flow depth at outlets	(m)
	Ev	*e*_*V*_	Vertical scale factor	(-)
	Diff_Q	$\Delta Q$	Difference between total inflow discharge and total outflow discharge^(5)^	(%)
Results	hA, hB, hC ^(1)^	*h*_*A*_, *h*_*B*_, *h*_*C*_	Time-averaged flow depth at each inlet^(3)^	(m)
	hA_i, hB_i, hC_i	*h*_*A,i*_, *h*_*B,i*_, *h*_*C,i*_	Temporal flow depth at each inlet	(m)
	SD_hA, SD_hB, SD_hC	${\sigma }_{{h}_{A}}$, ${\sigma }_{{h}_{B}}$, ${\sigma }_{{h}_{C}}$	Standard deviation of flow depth at inlets	(m)
	Q1, Q2, Q3	*Q*_1_, *Q*_2_, *Q*_3_	Flow discharge at each outlet	(m^3^h^−1^)
	QR1, QR2, QR3	*Q*_*R*1_, *Q*_*R*2_, *Q*_*R*3_	Flow discharge partition at outlets^(6)^	%
	h_Q1, h_Q2, h_Q3	*h*_*Q1*_, *h*_*Q2*_, *h*_*Q3*_	Time-averaged flow depth measured at each measurement channel	(m)
	h_Q1_i, h_Q2_i, h_Q3_i	*h*_*Q1,i*_, *h*_*Q2,i*_, *h*_*Q3,i*_	Temporal flow depth observed at each measurement channel^(4)^	(m)
	SD_Q1, SD_Q2, SD_Q3	${\sigma }_{{Q}_{1}}$, ${\sigma }_{{Q}_{2}}$, ${\sigma }_{{Q}_{3}}$	Standard deviation of outflow discharge^(4)^	(m^3^h^−1^)
	SD_QR1, SD_QR2, SD_QR3	${\sigma }_{{Q}_{R1}}$, ${\sigma }_{{Q}_{R2}}$, ${\sigma }_{{Q}_{R3}}$	Standard deviation of flow discharge partition^(4)^	(%)
	SD_h_Q1, SD_h_Q2, SD_h_Q3	${\sigma }_{{h}_{Q1}}$, ${\sigma }_{{h}_{Q2}}$, ${\sigma }_{{h}_{Q3}}$	Standard deviation of flow depth at measurement channels^(4)^	(m)

^(1)^The number of inlets and outlets varies with the model geometry. For each model, only the relevant variables were measured (e.g., only *h*_*B*_ and *Q*_*1*_, *Q*_*3*_ are available in the dataset for the bifurcation model).

^(2)^Measured at the location of the model boundary, see blue crosses in Fig. 3.

^(3)^Measured at the locations of green crosses shown in Fig. 3.

^(4)^In the cases where the outflow discharge is measured with the ‘volume filling’ method, time series of flow depths in the measurement channels and corresponding standard deviation are not available in the dataset. The same applies for the standard deviation of the flow discharge and discharge partition.

^(5)^: Δ*Q* = [(*Q*_*1*_ + *Q*_*2*_ + *Q*_*3*_) − (*Q*_*A*_ + *Q*_*B*_ + *Q*_*C*_)]/(*Q*_*A*_ + *Q*_*B*_ + *Q*_*C*_)

^(6)^: *Q*_*R,i = *_*Q*_*i*_ /(*Q*_*1*_ + *Q*_*2*_ + *Q*_*3*_), with *i* = 1, 2, 3.

Three files <file name> .py are available for reading the data in “<*file name>*.h5” file for the junction, the bifurcation and the district model.

For the third part (Dataset 2), the following files are available for each of the eight urban configurations presented in Fig. 4:

- “*<Config_XXX>.h5*” in HDF5 format. Besides the variables listed in Table 2, the file also contains time-averaged surface flow velocity for each configuration (V, Vx, Vy);
- <*Config XXX>*_data_structure.txt describes the corresponding HDF5 file;
- <LSPIV_VideoData_*ConfigName*>.zip provides data used for surface velocity measurement, including a series of recorded raw videos from several viewpoints, the coordinates of GRP points for each video, the parameters used for processing the videos with Fudaa-LSPIV for each sub-section. For more details, see the data description file (LSPIV-ParameterDescription.pdf) in the data package^18^.

### Notations and units

Datasets in the HDF5 files are labelled following the corresponding physical names. The names of the variables and the associated units are listed in Table 2.

## Technical Validation

### Accuracy of experimental boundary conditions

**Fig. 8:**
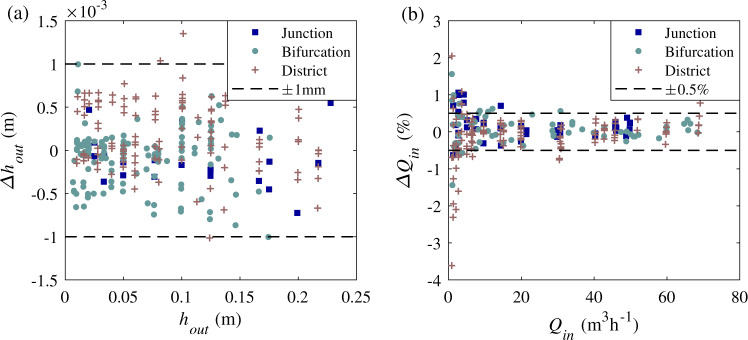
(**a**) Difference between measured and ‘target’ flow depths at model outlets; (**b**) difference between injected and ‘target’ flow discharges at model inlets. The dashed lines indicate the measurement uncertainties.

The quality of boundary condition settings is crucial for quantifying the effect of model geometric distortion^9^. Therefore, great efforts were made to prescribe accurately the ‘target’ values of inflow discharges and flow depths at the outlets. Fig. 8 shows the differences between measured and ‘target’ values of the boundary conditions. The biases for the flow depths and inflow discharges are generally lower than 1 mm and 0.5%, respectively. This is of the same order as the instrument measurement accuracy: 1 mm for the ultrasonic sensors and 0.5% for the flowmeters. Besides, Li et al. (2021)^9^ demonstrated that the outflow discharges are very sensitive to the downstream boundary conditions (in bifurcation and district models). Nevertheless, the trends in measured and computed upstream flow depths and outflow discharge partition are in good agreement when the model geometric distortion is varied. This suggests that the boundary conditions were indeed carefully prescribed in the laboratory tests.

### Flow depth measurements

**Fig. 9:**
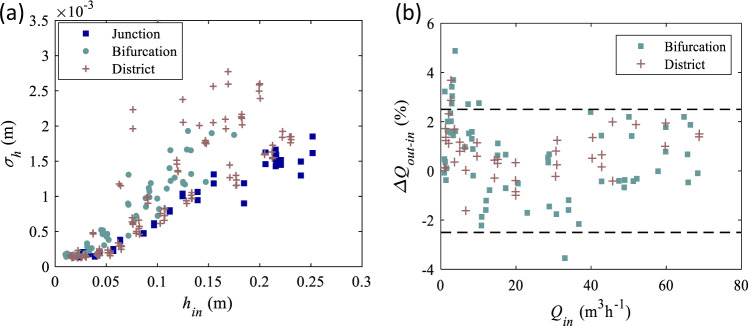
(**a**) Standard deviation of measured flow depth at the inlet of each model (junction, bifurcation and district model) as a function of the flow depth value; (**b**) Difference between the total injected inflow discharge and the total outflow discharge estimated by rating curves.

The accuracy of flow depth measurements is affected not only by the measurement uncertainty of ultrasonic sensors ± 1 mm but also by fluctuations of the free surface. Therefore, flow depth data were recorded for a duration ranging between 60 s and 150 s, depending on the cases, and time-averaged values were computed to reduce the influence of the fluctuations. The standard deviations of the time-series are of the order of 0.5 to 2 mm, as shown in Fig. 9a. To assess the reproducibility of the tests, measurements were repeated, and the observed differences did not exceed 1 mm, as detailed by Li et al. (2021a)^12^.

### Flow discharge

**Fig. 10:**
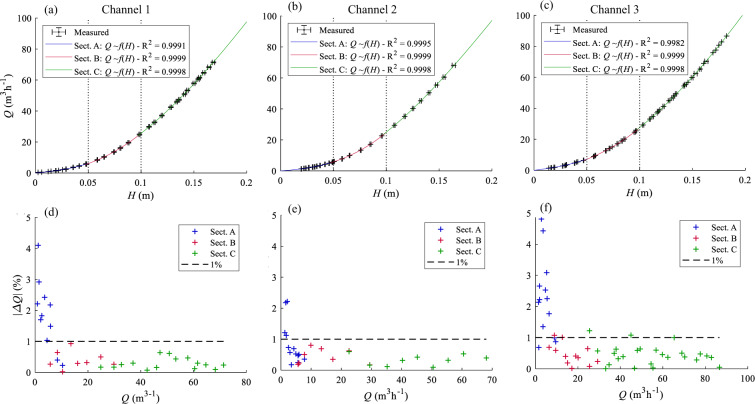
Rating curves linking the outflow discharge* Q* to the hydraulic head H measured in the measurement channels corresponding to outlet 1 (**a**), outlet 2 (**b**) and outlet 3 (**c**). Horizontal whiskers represent the standard deviation of time-series of recorded flow depths, vertical whiskers represent the standard deviation of time-series of flow discharges (obtained from the flowmeter). Relative errors between the measured values and values estimated from the rating curves, for outlet 1 (**d**), outlet 2 (**e**), and outlet 3 (**f**).

The uncertainty in the estimation of outflow discharges depends on the quality of the considered rating curves, and on the uncertainties affecting the determination of flow depths in the measurement channels. Fig. 10 shows the calibrated rating curves obtained for three measurement channels (blue, red and green lines correspond to subsections A, B and C, as depicted in Fig. 5). The agreement between the regression and the data points is characterized by R^2^ > 0.99. The relative error $| \Delta Q|$ between the measured values and the values calculated with the calibrated rating curves are generally below 1%, except for particularly small discharges (<3 m^3^h^−1^), for which this error can reach up to 5%. In such cases during experiments, the discharge was also measured manually (volume filling rate of a bucket) to minimize the measurement uncertainty related to the quality of rating curves. The uncertainty related to the measurement of flow depth in the measurement channels remains as low as described above.

The overall plausibility of flow discharge measurements was verified by mass balance analysis. Except for tests with very small total inflow discharges (mostly below 10 m^3^h^–1^), the differences between the total inflow and total outflow discharges does not exceed 2.5%, as shown in Fig. 9b.

### Surface velocity

A time convergence analysis was first carried out to determine the required recording duration (details in LSPIV data package^18^). A duration of 60 s provides a good compromise between the computational cost and the quality of obtained time-averaged velocities (i.e. the difference between the velocity field averaged over durations of 60 s and 90 s is lower than 0.01 ms^−1^ for more than 90% of measured points).

The whole surface velocity field was constructed by combining the results of several sub-regions. At locations where movies obtained from the different viewpoints overlap, the computed velocity fields show a good agreement and they were averaged (details in LSPIV data package^18^). Despite the advantage of the LSPIV technique, it remains challenging to precisely measure the flow velocity close to the sidewalls (in a narrow band of about 1 cm in width). Therefore, the velocities in these areas are not included in the datasets.

### Data usage caution

The outflow discharge in the junction model was not measured as it is equal to the sum of the inflow discharges in the main and lateral branches.

The standard deviation of inlet discharge for the tests with the junction model is not available due to a technical problem with the flowmeters during the corresponding series of experiments.

NaN values are displayed in the matrix of surface velocity in regions too close to the sidewalls (i.e. at a distance below 1 cm from the sidewalls). Since the spatial resolution of the measurement grid equals 1 cm, the dataset provides 18 measurement points of surface velocity over the crosswise direction of the main streets.

## Usage Notes

This dataset firstly aims to bring insights into the influence of the model geometric distortion and urban forms on flow depth and the discharge partition in laboratory modelling of urban flooding. It provides a rich dataset of flow hydrodynamic characteristics of urban flooding over a street network, which is essential for validating numerical models commonly used for estimating urban flood hazard. Researchers can also use the dataset to validate analytical hydraulic models.

## Data Availability

Algorithms for data processing were coded with Matlab 2018b, and the flow surface velocities were computed with the open-source software Fudaa-LSPIV (version 1.7.3). For further details, see https://forge.irstea.fr/projects/fudaa-lspiv.
